# A Personalized Approach to Managing Patients With an Ileal Pouch-Anal Anastomosis

**DOI:** 10.3389/fmed.2019.00337

**Published:** 2020-01-29

**Authors:** Zaid S. Ardalan, Miles P. Sparrow

**Affiliations:** Department of Gastroenterology, The Alfred Hospital, Monash University, Melbourne, VIC, Australia

**Keywords:** IPAA, carp, pouchitis, prophylaxis, ileoanal pouch, probiotic, prebiotic, surveillance

## Abstract

Quality of life after ileal pouch-anal anastomosis (IPAA) surgery is generally good. However, patients can be troubled by pouch-related symptoms and pouch disorders that can be inflammatory, mechanical/surgical, and functional. Management of patients with IPAA begins with measures to maintain a healthy pouch such as optimizing pouch function, providing tailored advice on a healthy diet and lifestyle, screening for and addressing metabolic complications of IPAA, pouch surveillance, and risk stratification for risk of pouchitis and pouch failure. Pouchitis is the most common inflammatory disorder. Primary pouchitis is a spectrum currently classified into three progressive phases—an antibiotic-responsive, an antibiotic-dependent, and an antibiotic-refractory phase. It is predominately microbially mediated in acute antibiotic-responsive pouchitis and predominately immune mediated in chronic antibiotic-refractory pouchitis (CARP). Secondary prophylaxis is recommended for recurrent antibiotic-responsive and for antibiotic-dependent pouchitis. Secondary causes of antibiotic-refractory pouchitis should be ruled out before a diagnosis of CARP is made. CARP is best classified as primary sclerosing cholangitis associated, immunoglobulin G4-associated, and autoimmune. Primary sclerosing cholangitis-associated CARP can be treated with budesonide or oral vancomycin. Early recognition of immunoglobulin G4-associated pouchitis minimizes ineffective antibiotic use. Autoimmune CARP can be managed in a manner similar to UC. The current place of immunosuppressives in the treatment algorithm depends on availability and early access to biological agents. Vedolizumab and ustekinumab are the preferred first- and second-line biologics for autoimmune CARP owing to their efficacy, better side effect profile, and low immunogenicity and need for concomitant immunomodulatory therapy. Antitumor necrosis factor should be reserved for autoimmune CARP failing the above and for CD of the pouch. There are no guidelines for the surveillance of pouches for dysplasia. Incidence varies based on a patient's risk. Since incidence is low, a risk-stratified approach is recommended.

## Introduction

Restorative proctocolectomy with ileal pouch-anal anastomosis (IPAA) is the preferred surgical treatment for most patients with ulcerative colitis (UC) and familial adenomatous polyposis (FAP). Quality of life (QOL) after colectomy and IPAA is generally good (1, 2). However, patients with IPAA are at risk of pouch-related symptoms of increased frequency, dietary intolerances, urgency, and incontinence (1). Furthermore, patients are at risk of inflammatory, surgical or mechanical, and functional pouch-related disorders. Management of patients with an IPAA begins soon after pouch creation and ileostomy closure with measures to optimize pouch function, maintain a healthy pouch, risk stratify patients to guide primary and early secondary prophylaxis for pouchitis, and ensure routine screening and monitoring for metabolic complications of the pouch. Furthermore, it is essential to have a thorough personalized approach for the various inflammatory, surgical/mechanical, and functional pouch-related disorders. Finally, some patients with IPAA are at risk of dysplasia and adenocarcinoma of the pouch. Knowing which patients are at risk and how best and how frequently to survey them is important. To ensure these various aspects of care are adequately delivered, it is recommended that IPAA patients continue to be managed in high-volume centers with multidisciplinary inflammatory bowel disease (IBD) or pouch clinics. Our IBD clinic manages ~1,250 IBD patients, which includes a growing number of IPAA patients at a rate of 10 IPAAs performed annually. This review outlines the principles of diagnosing and managing IPAA patients, with a focus on how to risk stratify and personalize management decisions in individual patients.

## Anatomy of the Ileoanal Pouch

An ileoanal pouch is created from 2(J), 3(S), or 4(W) limbs of the small intestine. Of these three pouch designs, the J pouch is the most popular owing to the ease of its creation and reliability of its function. S pouch has the advantage of an additional 2–3 cm of small bowel that can be connected to the anorectal transition zone, reducing anastomotic tension, and improving blood supply in those with a short mesentery. However, suboptimal evacuation and more challenging construction have led to it largely being replaced by the J pouch. The W pouch has largely been abandoned.

## Optimizing, Maintaining, and Monitoring the “Healthy” Pouch

### Optimizing Pouch Function and Maintaining a “Healthy” Pouch

Quality of life following IPAA surgery is generally good and, in some studies, reported to approach that of the general population at 12 months (3). Indeed, pouch function with reduced frequency and increased consistency continues to improve over the first 6–12 months as the pouch adapts. At 1 year, the accepted normal average bowel frequency is five to six during the day and one to two overnight. It is important that patients are educated about this adaptation period and the new “normal average bowel function,” particularly if the underlying indication for surgery was FAP or colitis-associated neoplasia where no or minimal symptoms existed before IPAA surgery. Furthermore, patients should be educated about dietary and pharmacological measures that can help improve pouch frequency and consistency.

Antidiarrheal medications such as loperamide, diphenoxylate/atropine, and codeine can be used to help reduce pouch frequency. Evidence supporting their efficacy is sparse. Loperamide is most widely used and, at a dose of 8 mg/day, has been shown to reduce pouch frequency and total stool weight (3).

Supplemental fibers like psyllium husk are frequently prescribed by colorectal surgeons to reduce frequency and improve stool consistency. Psyllium husk is a water-soluble fiber that is minimally fermentable. The reduced frequency and increased stool consistency can be explained by its effects on slowing upper gastrointestinal transit and increased stool bulk through water trapping. However, tolerability and efficacy of supplemental and dietary fibers are not universal among patients with some paradoxically developing loose stools and bloating. This could be related to the amount of fiber and associated small intestinal bacterial overgrowth (SIBO) (4). We recommend a trial of water-soluble minimally fermentable supplemental fibers such as psyllium husk in symptomatic patients who have an adequate intake of dietary fibers, starting at the smallest dose, increasing it in those who show partial response, and stopping in those who develop paradoxical worsening of symptoms or diarrhea.

There is currently no standardized dietary advice for IPAA patients. Observational studies suggest that most IPAA patients have at least one intolerable dietary substance negatively impacting pouch function (5, 6). However, there seems to be significant intersubject variability in what food type is intolerable (5). Therefore, a generalized dietary recommendation is not easy. One of the few products consistently shown to increase pouch frequency are caffeine-containing products (7, 8). A useful recommendation is not to exceed a cup or 250 g of a caffeine-containing product a day. Beyond such a recommendation, it is difficult to generalize dietary advice. Most patients end up following an individualized dietary habit through trial and error. A physician's main role is to ensure that the patient's diet has an adequate nutritional content, can optimize pouch function, and promotes a healthy pouch microbial community. Most diets adapted have adequate nutritional intake. Helping patients follow a diet that optimizes pouch function and promotes a healthy pouch microbial community can be challenging. Diet is the predominate factor that shapes the microbiota structure and function. This effect is mainly via dietary fibers and poorly digestible carbohydrates available for bacterial fermentation. A diet adequate in fermentable fibers is therefore central to achieving a healthy microbiota spectrum. However, readily fermentable fibers as fructooligosaccharides, inulin, and soluble non-starch polysaccharides, found in vegetables and fruits, induce an increase in pouch microbial mass and gas production, both of these factors contributing to increased stool bulk, reduced consistency, and increased frequency. Furthermore, the higher incidence of small intestine bacterial overgrowth in IPAA patients leads to more bacterial fermentation in the small bowel and release of gas causing bloating (4). Therefore, a more pragmatic approach is to try and achieve a balanced intake of fibers.

In addition to meal contents, meal volume, frequency, and timing influence pouch frequency. One study demonstrated a positive correlation between meal volume, meal frequency, and late night meals and pouch frequency, recommending no more than three meals a day with the last at least 2 h before bedtime (5).

### Preventing, Screening for, and Diagnosing Metabolic Complications of IPAA

Patients with healthy and inflamed IPAAs have a higher risk of iron deficiency anemia (IDA). Other causes of anemia include B12 deficiency, which has been reported in up to 25% of pouch patients (9). Patients with IPAA also have a higher incidence of low vitamin D and serum calcium independent of pouch inflammation (10). Vitamin D deficiency has been reported in 10–68% of patients (11). Bone loss is common in IPAA. Risk factors include old age, low BMI and pouchitis, primary sclerosing cholangitis (PSC), pouch villous atrophy, and lack of calcium supplementation (12). We recommend a baseline bone mineral densitometry in all patients. We also recommend calcium and vitamin supplementation in those with low levels of vitamin D or calcium, risk factors for, or confirmed, osteopenia.

### Risk Stratification and Prophylaxis for Pouchitis

The risk of developing pouchitis, the most common disorder of IPAA, varies among patients. Numerous risk factors have been identified. Assessing for the presence or absence of these risk factors can help guide the need for primary and secondary prophylaxis for pouchitis and manage patient expectations. Some risk factors such as the NOD2/CARD15 mutation (13) and certain Toll-like receptor genotypes (14) are costly, not widely available, and not routinely performed. We instead recommend focusing on risk factors that can be routinely assessed in clinic, providing a pragmatic risk stratification strategy.

- Primary Sclerosing Cholangitis (PSC): A positive association between pouchitis and PSC has been reported in numerous studies. The cumulative incidence of acute pouchitis at 10 years has been reported to be 70–80% (15, 16). Most studies have reported a higher incidence of chronic pouchitis among PSC ranging between 50 and 60% (15, 17, 18).
- Extraintestinal manifestations (EIMs): EIMs are a risk factor for acute and chronic pouchitis (19, 20). In one study, patients with precolectomy EIMs had a higher incidence of pouchitis compared to those with no EIM (39 vs. 26%, *P* < 0.01) (19). *De novo* EIMs post-IPAA are associated with an even higher risk of pouchitis (19). EIMs are also associated with a risk for chronic pouchitis with an odds ratio of 2.69; *P* = 0.047 (20).
- Concomitant autoimmune disorders: Unsurprisingly, “the presence of at least one autoimmune disorder is associated with a 2-fold risk of chronic antibiotic-refractory pouchitis (CARP)” (21). Immunoglobulin G4 (IgG4), a biomarker of autoimmune disorders, is associated with CARP. Antineutrophil cytoplasmic antibody is another serologic marker positively associated with chronic pouchitis with an odds ratio of 1.76; *P* < 0.01 in one study (22).
- Extensive colitis and backwash ileitis: The association of extent of colitis and back wash ileitis and acute and chronic pouchitis is unclear. Some studies have found extensive colitis to be a risk for acute and chronic pouchitis (23, 24). Others have found no association (25, 26). Backwash ileitis was shown in one study to be associated with increased pouch mucosal permeability (26). This is supported inconsistently by studies showing a positive association between backwash ileitis and acute and chronic pouchitis (27, 28). The discrepancy in these results can partly be explained by the difference in sample size, median follow-up, and difference in definition of pouchitis. We consider back wash ileitis as a useful adjunctive risk factor to the overall risk of pouchitis, rather than an independent risk factor.
- Corticosteroid exposure before proctocolectomy: Steroid dependence and high monthly steroid dose (defined as ≥ 500 mg/month before colectomy) have been associated with acute and chronic pouchitis, respectively, possibly reflecting more aggressive underlying autoimmune disease (29, 30).
- Periproctocolectomy thrombocytosis: In a prospective study evaluating the clinical factors for the development of pouchitis perioperative thrombocytosis, defined as a platelet count of >450 × 10^9^/L, it was found on multivariate analysis to be an independent risk factor for chronic pouchitis (odds ratio, 3.1; *P* = 0.03) (29).
- Young age: A few studies have reported and association between younger age at UC diagnosis or IPAA surgery and acute and chronic pouchitis as well as severity of pouchitis. In one study, patients who developed pouchitis had an earlier onset of UC (22.6 ± 1.3 years of age) compared with those who did not develop pouchitis (27.9 ± 1.1 years of age; *P* < 0.005) (31). In a Japanese study, chronic pouchitis was positively associated with age at the onset of UC of <26 years (32). In the Cleveland Clinic Ileal Pouch Center, chronic pouchitis is diagnosed more in pediatric patients than in their adult counterparts (33).
- Sex: Male sex is associated with acute and chronic pouchitis (33). A shorter male mesentery does theoretically risk-reduced pouch perfusion. While this can explain the increased incidence of ischemic pouchitis in men, how this affects the pouch microbial community and mucosal immune response is not clear.
- Type of ileal pouch: Although harder to construct and with inferior pouch function, S pouches are significantly less likely to be complicated with CARP than J pouches (*P* < 0.001) (34).
- Postoperative non-steroidal anti-inflammatory drug use: Defined as more than 1 week of regular NSAIDs postoperatively, NSAID use has been associated with chronic pouchitis (20).
- Smoking status: The association of smoking and acute and chronic pouchitis is interesting. Smoking is known to have a protective effect in UC and a detrimental effect on the natural course of Crohn's disease (CD). The protective effect in UC is unclear, but smoking or nicotine reduces gut mucosal permeability and hence the antigen load triggering a mucosal immune response (35). Chronic antibiotic-refractory pouchitis is predominately immune mediated and is often compared to UC. Indeed, smoking has been negatively associated with CARP (29). The effect of smoking on acute antibiotic-responsive pouchitis is less clear. In two studies, a never-smoker status was a risk factor for all pouchitis (36). In another study, active smoking was positively associated with acute pouchitis (29). One possible explanation for the increased prevalence of acute antibiotic-responsive pouchitis in smokers is the effect of smoking on the microbiome, which is known to be crucial for mediating acute pouchitis (37).

Chronic antibiotic-refractory pouchitis, which is immune mediated, has several shared etiopathological risk factors. Patients with these risk factors have a primed immune system with a lower threshold for initiating and maintaining an abnormal mucosal immune response. Therefore, patients harboring one or more of these risk factors should be counseled about primary prophylaxis of pouchitis, as is discussed below. Similarly, those harboring one or more risk factors who have acute antibiotic-responsive pouchitis (<4 episodes of acute pouchitis a year) can also be counseled about early commencement of secondary prophylaxis.

### Primary Prophylaxis of Pouchitis

#### Probiotics

Probiotics are live microorganisms belonging to the gut flora that can be safely ingested to exert health benefits. Probiotics have been tried for primary prophylaxis of pouchitis in at-risk patients. The probiotic agent VSL#3 (*Lactobacillus* spp., *Bifidobacterium* spp., *Streptococcus salivarius* spp., and *Thermophilus* spp.) at a dose of 3 g/day was found in one randomized placebo-controlled trial of 40 patients to be associated with a lower pouchitis rate at 12 months (10%) compared with placebo (40%), *P* = 0.04 (38). In a separate randomized trial of 31 patients, there was no difference in the rate of pouchitis between those randomized to VSL#3 vs. placebo (39). *Clostridium butyricum* MIYAIRI, in a separate randomized trial of 17 patients, showed a trend toward less acute pouchitis compared to placebo (11 vs. 50% *P* = 0.14) over a period of 24 months (40). Although often of interest to patients, we acknowledge that the evidence base to support the use of probiotics for primary prophylaxis of pouchitis is not strong.

#### Antibiotics

There is paucity of research on the safety and efficacy of antibiotics for primary prophylaxis. In a small placebo-controlled randomized trial of 38 patients, tinidazole at a dose of 500 mg daily was associated with a lower rate of pouchitis at 12 months 19 vs. 58% in the placebo group, although it did not reach statistical significance (*P* = 0.21) (41).

#### 5-Aminosalicylates

There are no data on the efficacy of mesalazine in primary prophylaxis. The efficacy of sulfasalazine as a primary prophylactic agent was assessed in a retrospective case series where only 15% of the 20 patients on sulfasalazine (2,000 mg/day) developed pouchitis compared with 65% of the 31 controls at a median follow-up of 68 months (10–104) (42).

In conclusion, in patients with one or more risk factors for pouchitis, we recommend primary prophylaxis using probiotics. Since VSL#3 has the strongest available data, we recommend VSL#3 at a dose of 3 g daily. Other probiotics can be tried if VSL#3 is unavailable or costly. Alternatively, sulfasalazine can be used as the 5ASA of choice. We do not recommend using oral antibiotics as primary prophylaxis. This should be combined with dietary advice aimed at achieving a diet balanced in fermentable fibers to ensure a favorable microbial community.

### Secondary Prophylaxis of Pouchitis

The indications for and measures used in secondary pouchitis prophylaxis are discussed below.

## Evaluation and Management of Pouch-Associated Disorders

Pouch disorders can be classified as inflammatory, surgical/mechanical, and functional. Inflammatory disorders include pouchitis, cuffitis, and CD of the pouch. Surgical and mechanical disorders can be broadly divided into obstructive complications and leakage and fistula-related complications. Functional disorders include irritable pouch syndrome (IPS) and pelvic dyssynergia.

## Evaluation of the Ileoanal Pouch

**Figure 1:**
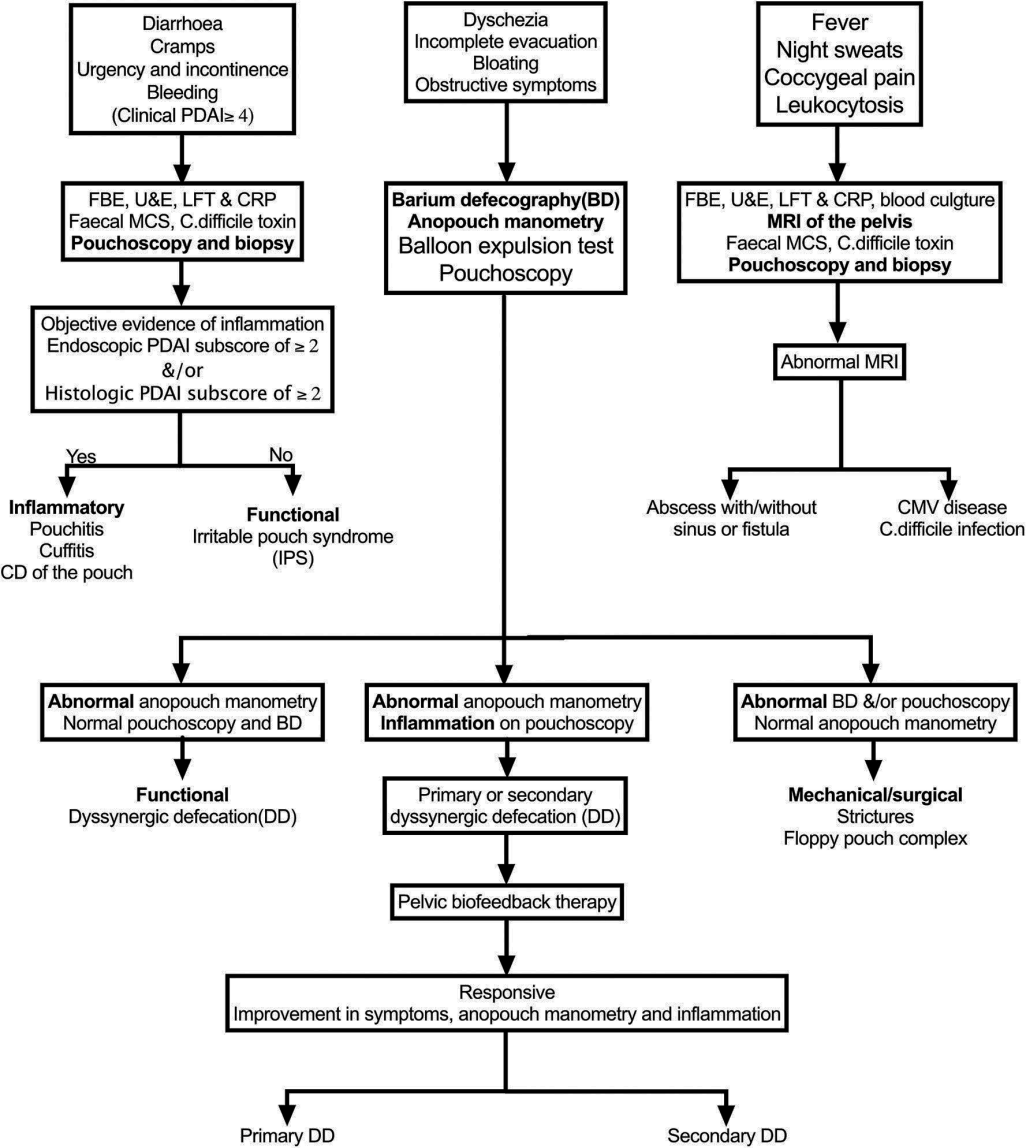
Algorithm for the evaluation of various pouch disorders based on the predominate symptoms. PDAI, Pouchitis Disease Activity Index; CD, Crohn's disease.

While laboratory tests are needed to investigate most pouch-related disorders, the most appropriate diagnostic test depends on the presenting signs and symptoms in an individual patient, as outlined in Figure 1.

- Diarrhea, cramps, urgency, and incontinence symptoms: pouchitis, cuffitis, CD, and IPS—best investigated with pouchoscopy and biopsy.
- Dyschezia, incomplete evacuation, bloating, obstructive symptoms: stricture, floppy pouch complex, and pelvic dyssynergia—best investigated with anopouch manometry and barium defecography.
- Fever, night sweats, coccygeal pain, leukocytosis: pathogens cytomegalovirus (CMV)/*Clostridioides difficile*, abscess, sinus fistula. CARP, cuffitis, and CD of the pouch rarely present with these symptoms—best investigated with fecal microscopy, culture, and sensitivity/*C. difficile* toxin and MRI of the pelvis.

### Diagnostic Tests Used to Evaluate IPAAs

- Pouchoscopy: Can be performed with a gastroscope or a colonoscope although we prefer the former. The three areas to examine include the prepouch ileum, the pouch body, and the cuff. A normal J pouch has an owl-eye appearance. Retroflexion is useful to assess the rectal cuff and essential if fistula is suspected. Biopsies should be taken from the three examined areas, biopsying away from suture lines.
- Imaging: The utility of cross-sectional imaging such as MRI or CT scan of the pelvis is mainly to investigate early and late mechanical or surgical complications as well as suspected perianal or peripouch complications of CD. Barium defecography is useful when investigating obstructive pouch-related disorders.
- Laboratory investigations:
  1. Bloods: Useful laboratory tests include full blood evaluation, urea and electrolytes, liver function tests, C-reactive protein. Patients with anemia should be further evaluated for underlying causes, especially iron deficiency anemia and B12 deficiency.
  2. Stool.
  - *Clostridioides difficile* toxin is particularly important in patients exhibiting fever or who are refractory to antibiotics.
  - Fecal calprotectin: There are limited data on the utility of fecal calprotectin as a non-invasive diagnostic tool for pouchitis. In a study of 54 patients with IPAA (46 UC and 8 FAP) who presented for routine pouchoscopy surveillance, fecal calprotectin was statistically significantly higher in patients with active pouchitis compared to those with inactive pouchitis. Receiver operating characteristic analysis demonstrated that a fecal calprotectin threshold of 92.5 μg/g was 80% sensitive and a 76.5 specific for the diagnosis of pouchitis [Pouchitis Disease Activity Index (PDAI) ≥ 7] (43). In another study of 60 patients with IPAA-UC, in the 10 patients (17%) who developed pouchitis, the median calprotectin was 112 μg/g. Importantly, calprotectin at a cut-off of 56 μg/g 2 months before patients became symptomatic of pouchitis had a 100% sensitivity and 84% specificity in predicting the episode of pouchitis (44). In a cross-sectional study of 32 UC patients who had had their IPAA created at the age of 12 ± 4 years, mean fecal calprotectin was 71 ± 50 μg/g among patients who have never had pouchitis (*n* = 10), 290 ± 131 μg/g among patients who have had at least one episode of pouchitis (*n* = 15), and 832 ± 422 μg/g among patients who have recurrent episodes of pouchitis (≥ 4 episodes/year) (45). We can conclude from these studies that fecal calprotectin is a practical and non-invasive investigation for symptomatic IPPA patients; however, the optimal threshold to diagnose pouchitis remains to be determined.
- Functional investigations: Anopouch manometry, balloon expulsion test, barium, or MR defecography are all investigations used to investigate patients with chronic dyschezia and are detailed below.

## Management of Pouch-Related Disorders

### Inflammatory Disorders

#### Pouchitis

Inflammation of the pouch is the most common pouch-related disorder with around 50–60% of UC patients and 20% of FAP patients suffering at least one episode at 10 years, a fifth of whom go on to develop chronic pouchitis (1, 46). A useful way to classify pouchitis is to divide it into primary and secondary pouchitis.

##### Primary pouchitis

**Figure 2:**
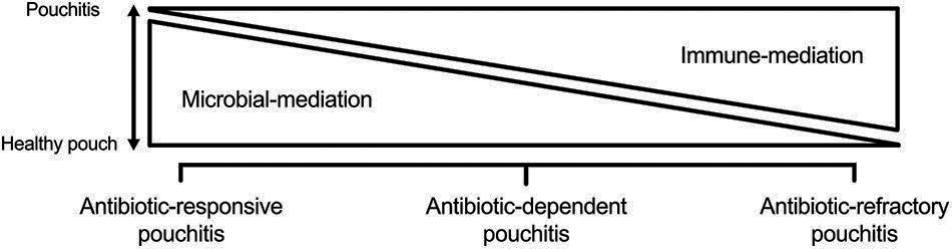
Primary that is predominately microbially mediated in antibiotic-responsive pouchitis and predominately immune mediated in chronic antibiotic-refractory pouchitis. Antibiotic-dependent pouchitis is somewhere in between.

This is defined as idiopathic inflammation of the pouch. Although etiopathogenesis is not completely understood, it is believed to be an abnormal immune response to some aspect of the pouch microbiome. It appears that early on, inflammation is largely microbially mediated as evident by the efficacy of antibiotics. Over time, inflammation can become predominately immune mediated, necessitating the addition of immunosuppressants. The pathogenesis of pouchitis and its subtypes are outlined in Figure 2. This could also explain the reduced frequency, delayed onset, and milder form of pouchitis in patients with FAP, whose immune system is not as “primed” as those with underlying UC (46). Primary pouchitis can further be classified according to the number of episodes of pouchitis and response to antibiotics into acute antibiotic responsive (<4 episodes a year), chronic antibiotic-dependent (4 or more antibiotic-responsive episodes or need for ongoing antibiotic use), and CARP, which is largely immune mediated. There are several diagnostic indices to assess inflammation of the pouch. The most widely used is the 18-point PDAI, which consists of symptom (0–6 points), endoscopy (0–6 points), and histology (0–6 points) subscores, as is outlined in Table 1. A total PDAI score of ≥ 7 points is considered diagnostic for pouchitis (47). A modified score, the modified pouchitis disease activity index (mPDAI), which omits histology, was suggested as an equally accurate alternative for the diagnosis of pouchitis with a score of ≥ 5 (48). The PDAI endoscopy score has six components (edema, granularity, loss of vasculature, friability, mucus exudate, and ulceration). One randomized controlled trial showed that the six components equally “contributed” to the total endoscopic score (48). However, recently, the appropriateness and reliability of each of the individual endoscopic components of the PDAI and other available diagnostic instruments, like the Heidelberg pouchitis disease activity index, was reassessed. Subsequently, the authors proposed removing edema, granularity, loss of vascularity, and mucus exudates as they were believed to be either inappropriate endoscopic features or of uncertain appropriateness with moderate interrater reliability. Ulceration, erosions, and bleeding were considered appropriate, with only ulcerations reaching substantial interrater reliability (49). Until these newly proposed criteria are verified, we suggest using the total PDAI score, including histological components. The histological subscore is composed of acute inflammatory changes such as neutrophil infiltration, crypt abscesses, and ulceration, seen on a background of chronic inflammation characterized by some degree of villous atrophy. A histological subscore of at least 2 is needed for a diagnosis of pouchitis. The PDAI is outlined in Table 1 (50).

**Table 1:** Pouchitis disease activity index (PDAI)^a^.

	**Score**
**CLINICAL**	
**Stool frequency**	
Usual postoperative stool frequency	0
1–2 stools/day > postoperative usual	1
3 or more stools/day > postoperative usual	2
**Rectal bleeding**	
None or rare	0
Present daily	1
**Fecal urgency or abdominal cramps**	
None	0
Occasional	1
Usual	2
**Fever (temperature** **>** **37.8°C)**	
Absent	0
Present	1
**CLINICAL SCORE**	/6
**Endoscopic inflammation**	
Edema	1
Granularity	1
Loss of vasculature	1
Mucopurulent exudate	1
Friability	1
Ulceration	1
Endoscopic score	/6
Acute histological inflammation	
**Polymorphonuclear inflammatory infiltrate**	0
Mild	1
Moderate + crypt abscesses	2
Severe + crypt abscesses	3
**Ulcers per lower power filed (%)**	0
<25	1
25–50	2
>50	3
Maximal acute histological inflammation	/6

a *Sandborn et al. (47)*.

##### Secondary pouchitis

Around 25% of chronic pouchitis are secondary to underlying conditions that need to be investigated and ruled out before a diagnosis of CARP is made. These include the following:

- Ischemia: Ischemia is one of the most common causes of secondary chronic pouchitis. It is characterized by asymmetric inflammation of the pouch involving the distal half, the afferent limb, or staple line (51). Risk factors include male gender and weight gain, as the proposed etiology is mesenteric tension. Ischemic pouchitis can be very challenging to treat. A trial of hyperbaric oxygen can be tried if available. In those with morbid obesity, bariatric surgery with consequent weight loss can reduce mesenteric tension and improve blood supply (52). Biological agents such as vedolizumab are recommended by The Cleveland Clinic Pouch Center, but outcomes have not been published.
- Crohn's disease of the pouch: The actual incidence of CD of the pouch is not known. In one study, 48 of 164 (28%) of patients initially diagnosed as having UC were diagnosed with CD upon reviewing their colectomy specimen before creating an IPAA (53). A two- or three-stage IPAA allows examination of the colectomy specimen for transmural inflammation or granulomas before an IPAA is created. However, CD of the pouch can occur *de novo*. The risk of *de novo* CD of the pouch in patients diagnosed with UC preoperatively is ~6% (54) and in those diagnosed with indeterminate colitis preoperatively is 15–20% (55). Known risk factors include a young age at diagnosis of UC (<20 years) and young age of surgery, indeterminate colitis, patchy colitis on colectomy specimen, active smoking, family history with CD, and seropositive anti-*Saccharomyces cerevisiae*-IgA (36, 56, 57). CD of the pouch can manifest as one of three predominate phenotypes, inflammatory, fibrostenotic, and fistulizing.
  a) Inflammatory CD of the pouch results in chronic pouch inflammation that may be associated with prepouch ileitis (PI) and deep ulcers in the pouch that is refractory to combination antibiotics for 4 weeks.
  b) Fibrostenotic CD results in ulcerated strictures anywhere in the jejunum, ileum, pouch inlet, or mid-pouch, associated with inflammation and/or ulcers of the afferent limb in the absence of NSAID use.
  c) Fistulae attributed to CD are non-anastomotic, developing at least 6 months after ileostomy closure in the absence of postoperative complications such as pelvic sepsis, leaks, or sinuses.
  The diagnosis of CD currently rests on a combination of clinical, endoscopic, histological, and radiological features. Fibrostenotic and fistulizing CD presenting in the fashion described above can usually be diagnosed endoscopically and radiologically. Crohn's disease presenting with chronic pouchitis can be harder to diagnose and distinguish from primary CARP, especially given that granulomas are only seen in 12–13% of cases, and transmural inflammation on radiological assessment is seen in both CD and CARP (1). The importance of distinguishing CARP from CD of the pouch lies in guiding the choice of biologic as antitumor necrosis factors (anti-TNFs) are more effective in those with CD (58) of the pouch compared to CARP patients who show better response to vedolizumab and ustekinumab (59, 60).
- Infections: CMV and *C. difficile* infection. The presence of fever should raise the suspicion of CMV and *C. difficile* infections.
  a) *C. difficile* infection (CDI) is a common cause of secondary pouchitis reported in as many as 18% of patients (61). Oral vancomycin should be considered first line in the management of pouch CDI. Recommended dose of oral vancomycin is 500–1,000 mg/day for 2–4 weeks. In patients with mild acute CDI who are metronidazole naive, oral metronidazole 500 mg twice daily for 2 weeks may be used as an alternative first line. Oral fidaxomicin 400 mg/day for 10–14 days or fecal microbiota transplantation are reserved for refractory or recurrent CDI (52, 62).
  b) CMV infection: CMV infection is rarely associated with pouchitis. The main risk factor is immunosuppression. On pouchoscopy, there is pouchitis and often ulcerating PI (63). Diagnosis should be based on the presence of CMV inclusion bodies or positive immune histochemistry. The presence of CMV PCR alone does not constitute a diagnosis of CMV pouchitis or require treatment. In one study, a positive CMV PCR was found in 41% of patients with antibiotic-responsive pouchitis that responded to conventional oral antibiotics (63). When therapy is considered, intravenous ganciclovir at a dose of 5 mg/kg every 12 h is the initial treatment of choice. In patients responding to IV ganciclovir, we recommend switching to an equivalent dose of oral valganciclovir−900 mg twice daily−2 days later to complete the 2- to 3-week course (63).
- Nonsteroidal anti-inflammatory drugs (NSAIDs): Regular use of NSAIDs postoperatively, defined as daily use of more than 1-week post-IPAA, has been found to be associated with acute and chronic pouchitis (36). Furthermore, patients on regular NSAIDs and pouch-related disorders benefit from complete discontinuation of these drugs, emphasizing the importance of inquiring about and stopping such agents in IPAA patients (64).
- Celiac disease: Celiac disease can develop *de novo* in patients with IPAA (65). Even if serology tests for coeliac were previously done and normal they should be repeated, and if positive, a duodenal biopsy should be performed to confirm the diagnosis.
- Once secondary pouchitis is ruled out a diagnosis of CARP, also referred to as immune-mediated pouchitis, is made. It is useful to classify CARP into PSC-associated CARP, IgG4-associated CARP, and autoimmune CARP; the management of each somewhat differs. The diagnosis of PSC is based on a magnetic resonance cholangiopancreatography, with or without a liver biopsy. The diagnosis of IgG4-associated pouchitis is confirmed by an elevated serum IgG4 with or without pouch and prepouch ileal infiltration with IgG4-positive plasma cells. Autoimmune CARP is simply CARP not associated with PSC or IgG4.

### Management of Primary Pouchitis

#### Acute Antibiotic-Responsive Pouchitis

First-line therapy includes a 2-week course of metronidazole (15–20 mg/kg/day) or ciprofloxacin (1,000 mg/day) (66). Ciprofloxacin appears to be more effective than metronidazole in treating active pouchitis, with fewer adverse effects (67). Tinidazole (1,000 mg/day or 15 mg/kg/day for 14 days) can be used as an alternative in those intolerant or failing the above and is considered one of the most potent agents here (52). In pregnant patients with pouchitis, amoxicillin-clavulanic acid may be safely used (52). Rifaximin 500 mg twice daily is also effective, but due to its cost and low side effect profile, it is best reserved for chronic antibiotic-dependent pouchitis requiring ongoing antibiotics (68). The efficacy of antibiotics suggests that some aspect of the pouch microbiome is injurious to the mucosa or triggers an immune response; therefore, attempts have been made to alter the microbiome or its metabolic output without the use of antibiotics.

Probiotics have been tried in acute pouchitis. High-dose VSL#3 at a dose of 3 g twice daily was found to be effective in a 4-week open-label trial (69), but a randomized controlled trial of 33 patients using a different probiotic showed no clinical, biochemical, or endoscopic response (70). Until there is further evidence to support their efficacy, probiotic agents are not recommended for the treatment of acute antibiotic-responsive pouchitis.

Dietary intervention is another potential alternative. In patients with IPAA, there is emerging evidence implicating the relative and absolute concentration of the microbial metabolites hydrogen sulfide (H2S) and butyrate, a short-chain fatty acid (SCFA), in the pathogenesis of pouchitis. Studies have shown an association between H2S production and the number and severity of pouchitis episodes (71). Reduced fecal butyrate has also been associated with pouchitis in a number of studies (72, 73). Since they are by-products of bacterial metabolism, H2S and SCFA production depends on the availability of dietary substrates. A diet which aims at increasing SCFA and reducing H2S can theoretically target the potential pathogenesis of pouchitis, but there exists no data supporting its tolerability or efficacy to date.

Patients failing to respond to 2 weeks of one of the antibiotics can be treated with the other agent for 2–4 weeks. Patients failing metronidazole should be treated with ciprofloxacin. Patients failing ciprofloxacin can be treated with metronidazole, although we prefer using tinidazole, as it appears to be better tolerated and more efficacious against potentially resistant microbes (74). Patients failing 4 weeks of monotherapy should be treated with 4 weeks of combination therapy. Combination therapy of ciprofloxacin and metronidazole for 4 weeks achieved remission in 82% of patients in an open-label study (75). Those intolerant to metronidazole can be treated with a 4-week course of ciprofloxacin and tinidazole (74) or a 2-week course of ciprofloxacin and rifaximin (76). Patients failing 4 weeks of combination therapy are considered to have CARP and need to be investigated for secondary causes of pouchitis. Those who do respond would benefit from the same measures used in patients with antibiotic-dependent pouchitis discussed below. In patients readily responding to first line antibiotics, secondary prophylaxis to prevent future episodes can be considered. This is particularly useful in those with the aforementioned risk factors, when episodes are recurrent or when approaching important life milestones such as marriage, having children, commencing a new job, or planning a vacation.

#### Chronic or Recurrent (Four or More Episodes) Antibiotic-Dependent Pouchitis

The etiopathogenesis of idiopathic pouchitis is better thought of as a spectrum, whereby it is predominately microbially mediated in antibiotic-responsive pouchitis and predominately immune mediated in CARP. Antibiotic-dependent pouchitis is somewhere in between (see Figure 2), with treatment measures aimed at the microbiome, with or without the addition of measures aimed at suppressing the mucosal immune response.

#### Addressing the Microbial Component

Any of the antibiotics used for the treatment of antibiotic-responsive pouchitis can be used at the lowest needed dose to maintain remission in antibiotic-dependent pouchitis. However, prolonged use of metronidazole and ciprofloxacin is associated with potential adverse effects such as peripheral neuropathy and tendinopathy, respectively. In a study that followed 39 patients with antibiotic-dependent pouchitis on metronidazole or ciprofloxacin for 1 year, adverse effects were reported in 11 (28%) patients, and antibiotic resistance was found in at least one stool sample of 28 (78%) patients (77). Rifaximin is an oral, broad-spectrum, minimally absorbed GI-specific antibiotic with no clinically significant bacterial resistance (78). In an open-label study of 51 patients with antibiotic-dependent pouchitis, rifaximin at a dose of 200–1,800 mg/day was used to maintain remission following a 2-week course with ciprofloxacin or metronidazole. At 3 months, 33 (65%) patients remained in remission. Of the 33, 19 (58%) remained in remission for 12 months (79).

Alternative approaches that bypass the need for antibiotics in antibiotic-dependent pouchitis through attaining and maintaining a healthy microbiota spectrum or microbiota function include (i) the use of probiotics, (ii) the use of the potentially healthy products of microbiota fermentation such as butyrate, or (iii) the use compounds that bind or inactivate the potentially harmful products of microbiota metabolism such as bismuth that binds H2S.

##### Probiotics

Probiotics have been tried for secondary prophylaxis in patients with antibiotic-responsive and chronic antibiotic-dependent pouchitis. Two early placebo-controlled randomized studies of 40 and 36 patients investigated the efficacy of VSL#3 at a dose of 6 g/day. In both, 85% of the treatment group maintained remission at 9 months compared to 0% in the placebo group (80, 81). However, postmarketing open-label studies and more recent randomized trials have been disappointing (82). The cause of these contradictory outcomes is uncertain. Various factors could potentially play a role in patients' response to probiotics such as host genetic or mucosal immunological factors, microbiota profiles, or probiotic composition or dose. In addition, it is not known whether patients' different dietary habits played a role in the different responses. A better understanding of how probiotics work could help choose the right probiotic composition and dose for the right host. One of the most common proposed mechanisms of probiotic benefits is suppression of resident pathogenic bacteria; however, in the randomized trial of VSL#3 use for primary prophylaxis that measured fecal cultures, VSL#3 was not associated with decreased fecal concentrations of *Bacteroides*, coliforms, *Clostridioides*, enterococci, or total aerobes and anaerobes in responders despite the increased fecal concentration of all eight strains of ingested bacteria, suggesting that protection was not mediated by “suppression of endogenous luminal bacteria” (80). Bifidobacterium are primarily acetate producers but also are primary degraders of fibers providing intermediates to most other saccharolytic bacteria. An increase in SCFA production following ingestion of *Bifidobacterium*-containing probiotic has not been assessed. Finally, an intriguing proposed mechanism is the induction of host-protective immune responses. Lactobacilli have been found to stimulate secretory immunoglobulin A, mucosal interleukin-10, and systemic Th2 responses (83). Understanding these mechanisms of action, the patient's microbiota structural and functional profile and what members of the bacterial community are responsible for the constant antigenic drive leading to Th2 cellular activation may, allow an individualized approach of targeted probiotic therapy.

##### Prebiotics

Fibers are preferentially fermented over protein by gut microbiota, increasing SCFA and reducing H2S, potentially reducing or preventing inflammation. Inulin was tried in a 3-week crossover randomized double-blinded placebo-controlled trial. It resulted in a statistical reduction in endoscopic and histological PDAI subscores (84). There were no differences in pouch microbiota on fecal cultures. In another crossover placebo-controlled study, 14.3 g of fructans (fructooligosaccharides) increased fecal butyrate and reduced protein fermentation while slightly increasing stool frequency from six to seven bowel actions a day. An equal amount of resistant starch increased fecal butyrate without changing protein fermentation, stool frequency, or weight (85). The combination of fibers and probiotics has also been tried. In a pilot study published only in abstract form, the combination of probiotic (*Lactobacillus* GG) and prebiotic (fructooligosaccharides) capsules resulted in complete resolution of symptoms and reversal of endoscopic and histological features in 10 patients with chronic antibiotic-dependent and antibiotic-refractory pouchitis (86). Furthermore, The Cleveland Clinic Pouch Center found that combining over-the-counter probiotics, as dietary supplements, with fibers in the form of tablets, capsules, suppositories, enemas, and foams resulted in a 3-fold rise in SCFA production in the ileal pouch, although tolerability and clinical efficacy was not reported (87). The efficacy of topical SCFAs in the ileal pouch by administering SCFA enemas has been tried in small uncontrolled studies. They have shown an overall minimal clinical response rate (88–90).

##### Bismuth

One approach that has been proven effective at reducing fecal H2S is the use of bismuth that binds sulfide in a dose-dependent manner (91). In healthy volunteers, a dose of 524 mg of bismuth subsalicylate (Pepto-Bismol^®^) four times daily (qid) resulted in 100-fold reductions in H2S release (92). The efficacy of bismuth has been tried in patients with CARP. In an open-label study, bismuth-citrate carbomer enemas were shown to be effective with 83% of patients entering remission. However, in a randomized trial of CARP patients, bismuth carbomer foam enemas nightly for 3 weeks were found to be ineffective (93). Oral bismuth subsalicylate at a dose of 250 mg three times daily or qid) was found to be safe, tolerable, and effective at improving symptoms in 85% of patients with CARP, allowing half of them (45%) to discontinue antibiotics after 4 weeks (94).

In conclusion, we have a number of measures available to address the microbial component in patients with antibiotic-dependent pouchitis. We recommend starting with probiotics recommending VSL#3 at a dose of 6 g daily given its reported efficacy and safety in earlier studies. If it is costly or unavailable, we would recommend trying an alternative probiotic containing Lactobacilli and Bifidobacteria. We recommend using oral antibiotics as a second-line maintenance agent due to their potential side effects, possible reduced long-term efficacy and cost. Rifaximin is our antibiotic of choice due to its safe side-effect profile. We use a dose of 500 mg daily, although any dose between 200 and 1,800 mg can be used. This strategy can by limited by rifaximin's high cost. We reserve ciprofloxacin (250–500 mg/day) or tinidazole (250 mg/day) for those who cannot obtain rifaximin or when it has failed. Owing to the side effects from long-term use, we would not recommend using ciprofloxacin or tinidazole continuously for more than 1 year. Oral bismuth subsalicylate at a dose of 250 mg (three times daily or qid) is used as third line following oral antibiotics. Until we have more robust data on the dose, efficacy, and tolerability of fibers and SCFA, we recommend combining all these measures with dietary advice on a diet rich in fermentable fibers, individually adjusting the quantity and type of fibers according to the patient's tolerability. Finally, since antibiotic-dependent pouchitis is both microbially and immune mediated, it is reasonable to add measures addressing the immune response to any of the above in an attempt to help patients remain in remission while discontinuing medications with potential side effects such as ciprofloxacin.

#### Addressing the Mucosal Immune Response

Patients partially responding to measures targeting the pouch microbiome or those on long-term antibiotics wanting to reduce or discontinue them can be treated with measures aimed at suppressing the mucosal immune response.

##### 5ASA

Topical and oral mesalazines have been tried in patients with CARP showing a 50% remission rate (74). Sulfasalazine at a dose of 2 g/day was investigated as a primary prophylaxis agent. Given their safety and tolerability profile, topical or oral 5ASAs can be tried in patients with antibiotic-dependent pouchitis to see if they can help reduce or discontinue antibiotic use (42).

##### Corticosteroids

Budesonide enemas at a dose of 2 mg/100 ml a day for 6 weeks were found to be non-inferior and more tolerable than metronidazole for the management of acute pouchitis (24). Oral budesonide was assessed in 14 patients with acute pouchitis (*n* = 6) and chronic pouchitis (*n* = 8) associated with PSC. Patients were treated with 9 mg/day of budesonide for 1–3 months and maintained on 3–6 mg/day for 9 months. At 1 year, 75% maintained remission including all of those with acute pouchitis and six of eight of those with chronic pouchitis. An 8-week course of oral budesonide controlled ileal release (9 mg/day) was also successful in inducing remission in 75% of patients with autoimmune CARP. The use of budesonide can therefore be tried, although more data on long-term efficacy and safety are needed before this is a standard recommendation.

#### Chronic Antibiotic-Refractory Pouchitis

In CARP, pouch microbiota may still play a role in driving inflammation, as evident by some response to antibiotics, but the disease is predominately immune mediated and is sometimes referred to as immune-mediated pouchitis. Therefore, it is best managed with medications that address the mucosal immune response. The classification of CARP into PSC-associated CARP, IgG4-associated CARP, and autoimmune CARP helps guide management.

**Figure 3:**
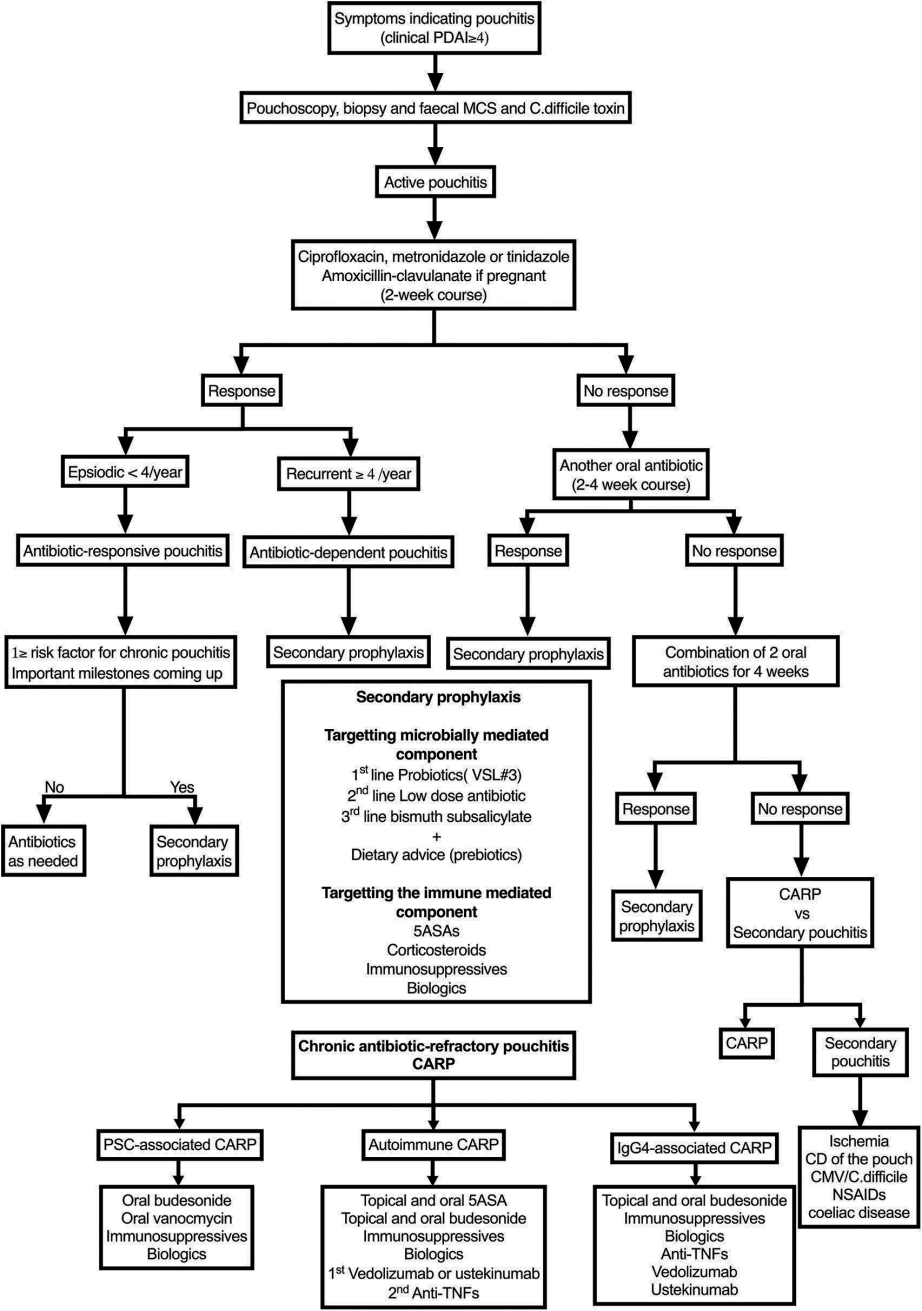
Algorithm for managing pouchitis. PDAI, Pouchitis Disease Activity Index; PSC, primary sclerosing cholangitis; NSAIDs, non-steroidal anti-inflammatory drugs; CARP, chronic antibiotic-refractory pouchitis.

a) PSC-Associated CARP
  - Budesonide: As detailed above, budesonide has been shown to be effective in inducing and maintaining remission in PSC-associated CARP (95), but the dose needed for long-term maintenance and its long-term efficacy and safety are yet to be determined.
  - Vancomycin: Oral vancomycin (500–1,000 mg/day) is successfully used to achieve and maintain remission in PSC-associated pouchitis/enteritis at the Cleveland Clinic Center for Ileal Pouch Disorders (52). We have had similar success inducing remission with oral vancomycin at a dose of 250 mg qid. Furthermore, vancomycin may provide an added benefit of improving liver function tests (96–98). There are no published data on the long-term efficacy or safety of oral vancomycin in IPAA patients. Most of the available data are from patients with recurrent CDI. These studies have not shown an increased risk of adverse events; however, they are limited by short duration of follow-up and lack of prospective, standardized follow-up to detect safety-related outcomes (99). Oral vancomycin has been shown to reduce bacterial richness and diversity and to increase the risk of vancomycin-resistant enterococcus colonization in patients with recurrent CDI. In PSC patients, oral vancomycin has been well-tolerated (97, 98). Therefore, the efficacy and potential hepatoprotective effect of oral vancomycin and the long-term efficacy and side effects of other immune suppressants should be weighed against the potential adverse effects of long-term vancomycin use. We recommend a trial of oral vancomycin to induce remission at a dose of 250 mg qid for 4–8 weeks followed by an attempt to maintain remission with a dose of 125 mg−250 mg qid. This can be tried before or after other immune suppressants used for non-PSC-associated pouchitis.
b) IgG4-Associated Pouchitis
  IgG4-associated pouchitis was first described by Shen et al. (100). This is an immune-mediated pouchitis often associated with a long segment of PI (101). Early recognition may help minimize antibiotic use and direct treatment to measures addressing the mucosal immune response early on. There are limited data on treatment options. There are no data on the efficacy of 5ASA or immunomodulators such as thiopurines and methotrexate. Corticosteroids such as budesonide have been reported to improve inflammation in case series (102). Patients failing budesonide should be considered for biological therapy. Unlike autoimmune CARP, the efficacy of different biological agents is not published. There are case reports of IgG4-mediated diseases (pancolitis and ocular adnexal disorder) responsive to adalimumab and infliximab (103, 104). Rituximab, a monoclonal antibody against CD20-positive lymphocytes, is used successfully in other IgG4-mediated diseases (105). We recommend using oral budesonide as first-line treatment starting with a dose of 9 mg for 8 weeks, then weaning it down to a maintenance dose of 3–6 mg daily. The next step is not clear. A step-up approach similar to that of autoimmune CARP can be followed, although vedolizumab and ustekinumab are not necessarily preferred over anti-TNFs. Rituximab can be considered in those failing other biologics and before pouch excision or diversion.
c) Autoimmune CARP
  The management of autoimmune CARP shares a great deal of similarity to that of UC.
  - 5ASAs: Topical and oral mesalazines Canasa^®^ suppositories (1,200 mg/day), Rowasa^®^ enemas (4,000–8,000 mg/day), and oral Pentasa^®^ (2,400–4,800 mg/day) have been tried in patients with CARP, demonstrating remission rates of 50% (74). Owing to their safety profile, oral or topical mesalamine agents are the preferred first-line drugs for autoimmune CARP.
  - Budesonide: As detailed above, topical budesonide enema 2 mg/100 ml and oral budesonide-controlled release 9 m/day have been shown to effective in inducing remission in acute and autoimmune CARP (95, 106). Budesonide enemas can be tried in those intolerant or failing 5ASAs. While oral budesonide may be useful in inducing remission particularly in those with associated PI, the dose needed to maintain remission and the long-term efficacy is not yet known. As such, ongoing use should be weighed against the long-term efficacy and safety of immunomodulators and biological agents.
  - Immunomodulators: Historically, immunomodulators including azathioprine (50–100 mg/day), 6-mercaptopurine (50–100 mg/day), and oral or subcutaneous methotrexate (7.5–25 mg/week) have been used as second-line therapy for autoimmune CARP, particularly in those with extra intestinal manifestations. There is, however, a paucity of data on the use of immunomodulator monotherapy for pouchitis (107). In contrast, there are more data supporting the efficacy of biological agents, particularly vedolizumab and ustekinumab, in the treatment of autoimmune CARP (59, 60). The current place of immunomodulators in the treatment algorithm, therefore, depends on availability and early access to biological agents.
  - Biological agents: To this date, no randomized controlled studies assessing the effectiveness of biological therapy for CARP exist. Most available data come from small observational studies.
    a) Vedolizumab: In the largest observational study, 20 patients with chronic, antibiotic-dependent, or refractory pouchitis were treated with vedolizumab using the standard IBD dose in 10 centers in Germany. At 14 weeks, the overall reported response rate (defined as a PDAI fall of 3 points or more) was 64% with a drop of median PDAI from 10 to 3 and discontinuation of antibiotics in 17 out of 19 patients. In addition, no serious side effects or intolerances were reported (59). Other case series have reported similar efficacy (108, 109).
    b) Ustekinumab: In the largest observational study, 24 patients with CARP (including 2 with PSC-associated CARP) were treated with ustekinumab using standard CD dosing. There was a 50% clinical and endoscopic response. The clinical response demonstrated was an improvement in median pouch frequency from 8 to 6 (*P* = 0.002). The endoscopic response was a decrease in ulcerated surface from >10 to <10% (60).
    c) Anti-TNF: In a systematic review, the short- and long-term efficacy of anti-TNF therapy (infliximab and adalimumab) in CARP were analyzed. Short-term efficacy was defined as clinical remission at week 8. Long-term efficacy was defined as clinical remission at the end of year. Short-term efficacy was 10%, and long-term efficacy was 37%. There was significant heterogeneity among the studies. For example, one study assessing the short- and long-term efficacy of infliximab on 24 CARP patients showed an 88% clinical response rate (14 partial, 8 complete) at week 10 with 56% maintaining this response at a 20-month median follow-up. In a more recent study, not included in the meta-analysis, the efficacy and tolerability of infliximab (*n* = 12) and adalimumab (*n* = 3) were assessed. At week 14, clinically relevant remission, defined as a mPDAI <5 and a reduction of mPDAI ≥ 2 points from baseline, was achieved in 43.5% of the infliximab group and 38.5% of the adalimumab group. In the long term, 40.7% discontinued anti-TNF therapy due to intolerance or drug reaction (109). We recommend using vedolizumab as the first-line biological therapy followed by ustekinumab owing to their efficacy, better side-effect profile, and low immunogenicity, and need for concomitant immunomodulatory therapy. We recommend reserving the use of anti-TNFs to those failing vedolizumab and ustekinumab. The management algorithm for pouchitis is seen in Figure 3.
  - Surgery: Surgery may be considered as a last resort for patients with CARP refractory to all medical therapy. The procedure of choice is an end ileostomy, with or without pouch excision. This should be reserved for patients with ongoing symptoms significantly impacting on QOL as stoma complication rates can be as high as 35–40% (110). Furthermore, the decision to remove the pouch or leave it *in situ* includes balancing a 35–40% of pouch stump sinus with pouch excision vs. a 50–60% of diversion pouchitis and other complications including pouch stricture dysplasia (110).

### Cuffitis

Cuffitis is defined as residual inflammation of the rectal cuff which will appear on pouchoscopy as 360° circumferential inflammation of the rectal cuff with histological findings consistent with UC proctitis. Patients are at a higher risk if there is a long-retained cuff > 2 cm. The treatment of cuffitis is similar to that of proctitis, starting with topical therapy with 5ASAs, corticosteroids, and escalating treatment to oral 5ASAs, immunomodulators, and eventually biological agents. As with proctitis, refractory cases can be treated with tacrolimus suppositories (111). Importantly, medically refractory cuffitis should raise suspicion for CD-associated cuffitis or pericuff fistula, sinus, or abscess. This can be further investigated with a pelvic MRI, contrast pouchogram, and examination under anesthesia. Furthermore, a foreign body, such as a retained suture in the anterior wall of the cuff, can cause local ulceration with significant urgency not responding to topical treatment. Diagnosis can be made on pouchoscopy with local ulceration at 3–5 o'clock with an underlying foreign body; treatment is surgical. Finally, idiopathic medically refractory cuffitis can be treated surgically. Redo IPAA is possible if there is a long-retained cuff. Another surgical treatment is cuff mucosectomy and pouch advancement (112).

### Crohn's Disease of the Pouch

At present, there are no guidelines on the best treatment approach for patients who develop CD of the pouch, and the risk of pouch failure with diversion or excision remains high at 17–57% depending on the series (55). The efficacy of various treatments are discussed comprehensively by Lightner et al., concluding that different treatment regimens may be effective based on phenotypic stratification, with fistulizing disease requiring the most aggressive treatment (55). Debilitating CD of the pouch refractory to all medical therapy should be discussed in a multidisciplinary team at a high volume center, as it may need surgical intervention in the form of permeant diversion or pouch excision. These procedures are associated with higher risks of complications and can be as challenging as IPAA surgery. The complication rate of permanent diversion or secondary ileostomy is 35–40% in addition to the risk of diversion pouchitis and pouch strictures (50–60%) precluding dysplasia surveillance. Pouch excision, on the other hand, is not without risks with a reported 35–40% risk of pouch stump sinus. Therefore, surgery should be reserved to CD patients failing all other treatment options (113).

### Prepouch Ileitis

Prepouch ileitis (PI) is defined as acute or chronic inflammation of the prepouch ileum extending in a contiguous fashion from the pouch inlet beyond 2 cm and up to as much as 30, and in one study 50 cm (114, 115). It manifests endoscopically as erosions, ulcers, erythema, friability, and strictures. It is usually associated with pouchitis (115, 116). The importance of recognizing and distinguishing PI from pouchitis alone is that PI appears to be an immune-mediated process not seen in FAP, is less responsive to antibiotic therapy, and is associated with a more severe course than pouchitis alone. Furthermore, PI needs to be distinguished from CD, which has a much higher rate of pouch failure. A diagnosis of CD can only be made when disease is more proximal, is segmental, includes deep fissures, includes fistulas, is associated with perianal disease, and the finding of “transmural lymphoid aggregates and epithelioid granulomas” (114). Moreover, CD manifesting as PI is less likely to be associated with pouchitis. Importantly, in IPAA patients with a history of indeterminate colitis, the diagnosis of PI strongly suggests CD or CD-like behavior with high pouch failure rate, and, therefore, a need for early aggressive medical and surgical therapy. Finally, the presence or absence of PSC or IgG4 should be determined as the PSC- and IgG4-associated PI should be managed like PSC- and IgG4-associated CARP, respectively. The management of PI associated with IPAA-UC follows the guidelines of managing idiopathic pouchitis. Since antibiotics have a 50% failure rate and the disease is predominately immune mediated, we recommend either commencing treatment with immunosuppressives or biologics or escalating rapidly to them. Immunosuppressive and biological treatments are the same as that used for autoimmune CARP. There is a small number of published studies on the efficacy of immunosuppressives and biologics, and they are largely observational and retrospective. Most data are with infliximab, with response rates ranging from 25 to 56% (114, 117). Accordingly, the decision on which immunomodulator or biologic to use should be individualized taking into consideration the patient's prior biological exposure, age, and infection and cancer risk.

## Surgical and Mechanical Pouch Disorders

A basic understanding of surgical and mechanical complications is useful when managing symptomatic pouch patients. This helps facilitate the most appropriate diagnostic test and the best effective treatment, be it medical or surgical. It is useful to broadly divide these disorders into obstructive and leakage-related septic complications. The complications, their risk factors, best diagnostic investigation, and recommended treatment are outlined in Table 2.

**Table 2:** Surgical and mechanical disorders of IPAA.

**Disorder**		**Risk factors**	**Incidence (%)**	**Presentation**	**Diagnosis**	**Treatment**
**OBSTRUCTIVE**						
Stricture	Stoma site	End to end anastomosis	5–11	Obstructive symptoms (abdominal pain, bloating, distention, incomplete evacuation)	Pouchoscopy MRE CTE	1st line: balloon dilatation. Needle knife for anastomotic structures in women. 2nd line: surgical stricturoplasty
	Inlet	Ischemia Anastomosis dehiscence Pelvic sepsis De-functioning ileostomy				
	Anastomosis					
Floppy pouch complex^a^	Pouch prolapses	Low BMI^**^ female sex	0.3	Obstructed defecation	Pouchoscopy (Collapse) BD^*^ (bulging) of the anterior pouch wall	Endoscopic banding. Surgery is ineffective.
	Pouch folding	Low BMI female sex	Unknown	Obstructed defecation	Pouchoscopy: pouch angulation. BD: C-shaped pouch	Surgical treatment
	Afferent limb syndrome	Low BMI female sex		Obstructed defecation Acute small bowel obstruction	BD: minimum contrast enters afferent limb	Surgical treatment
	Efferent limb syndrome	Long S-pouch efferent limb J-pouch with Long retained cuff (>7 cm)		Obstructed defecation Acute small bowel obstruction	Pouchoscopy: long cuff or efferent limb and angulation at body BD: similar findings	Surgical treatment Endoscopic balloon dilation of pouch inlet if surgery not possible or fails
**SEPTIC DISORDERS**						
Anastomotic^b^ leakage	Pelvic sepsis	Preoperative corticosteroid use Anastomotic tension Intra and post-operative blood transfusion Male sex BMI > 30	6–37	Postoperative sepsis	Laboratory blood tests Imaging: CT abdomen and pelvis BD	Antibiotics, percutaneous drainage, and surgical treatment
	Presacral sinus	Male sex Pelvic sepsis	5	Night sweats, fevers, tail bone pain, and weight loss	Pouchoscopy MRI of the pelvis BD	Endoscopic sinusotomy Pouch redo surgery
	Anastomotic fistula (Within 6 months post IPAA)	Pelvic sepsis 1 or 2 stage IPAA Female. sex: risks vaginal fistula	7	Draining fistula Pain and pelvic sepsis from an abscess	Pouchoscopy MRI of the pelvis EUA+	Surgical treatment

* *BD: Barium defecography*.

** *BMI: Body mass index*.

*+EUA: Examination under anesthesia*.

a *Khan and Shen (118)*.

b *Li et al. (119)*.

## Functional Pouch Disorders

### Irritable Pouch Syndrome

Around a third of patients with symptoms of frequency and urgency persisting beyond the 6- to 12-month adaptation period post-IPAA creation have no evidence of inflammation on laboratory tests or pouchoscopy (120). Using the total PDAI, these patients would have a score of <7 with a 0–1 pouchoscopy subscore. A diagnosis of IPS has been coined for these patients (120). There are no Rome criteria for the diagnosis of IPS; therefore, not all patients with symptoms of urgency and frequency may have IPS. Such a diagnosis, although not necessary, may offer reassurance and may guide management, as IPS therapy resembles that of IBS, starting with dietary modifications and then including use of antidiarrheals, antispasmodics, and even antidepressants (e.g., amitriptyline). Since there is significant intersubject variability on what food type causes symptoms, dietary modifications need to be personalized following a detailed review of the patient's dietary habits using a food frequency questionnaire and, if possible, a food dairy. Meal volume and frequency have also been shown to correlate with stool output; hence, meal frequency and volume should also be determined. Lactose intolerance can develop *de novo* after IPAA in some of patients. Poorly absorbed carbohydrates and fibers can be fermented by bacteria releasing gas and increasing stool bulk, exacerbating bloating, and pouch frequency. Indeed, most patients do report improved pouch symptoms of frequency urgency and bloating with a diet low in carbohydrates and fibers and high in meat. Interestingly, supplemental fibers like psyllium husk, frequently prescribed by colorectal surgeons, can reduce frequency and improve stool consistency in pouch patients when used in small amounts. These poorly fermentable fibers can slow the gastrointestinal transit and increase stool bulk through water-trapping effects. Therefore, use of poorly fermentable fibers can be tried particularly if bloating is not a predominate symptom. Some foods such as bananas, potatoes, pasta, and bread have been reported to decrease stool consistency or “thicken stools” and therefore may be tried to see if this helps reduce frequency (121, 122). Finally, one study found meal volume and frequency and late-night meals to correlate with pouch frequency, recommending no more than three meals with the last at least 2 h before bedtime (5).

Patients who have ongoing symptoms despite simple dietary modifications and a trial of fiber may benefit from a trial of the low fermentable oligosaccharides, disaccharides, monosaccharides, and polyols diet. This was found in a 6-week trial in 12 patients to improve median pouch frequency from eight to four in symptomatic patients with no pouchitis (123). Those whose symptoms persists despite dietary modifications and a low fermentable oligosaccharides, disaccharides, monosaccharides, and polyols diet can try antidiarrheal agents like loperamide or codeine or antispasmodics like hyoscyamine. If bloating is the predominate symptom, and since SIBO is common in patients with IPAA (4), a diagnostic and therapeutic trial of antibiotics used in SIBO can be tried. Finally, IPS is characterized by visceral hypersensitivity (23). Therefore, like IBS, neuropathic medications like amitriptyline can be tried at the dose used for IBS at 10–50 mg nightly.

It is important to note that some IPAA patients report no increased frequency or urgency and no obstructive symptoms. Instead, they are profoundly troubled by other symptoms such as seepage, nocturnal incontinence, daytime incontinence, and intense perianal burning. General advice provided to reduce seepage includes a small meal at least 3 h before bedtime, emptying the pouch at bedtime, and taking 4 mg of loperamide. The latter has been the only measure associated with improved sphincter continence (124). As sphincter strength decreases over time, daytime incontinence can affect up to 40–50% of patients after 20–30 years, causing significant distress and impacting on social life and QOL (125). The dietary measures discussed for frequency and urgency can be tried here, especially food types found to “thicken stools.” Fiber supplements can increase stool bulk, and loperamide can reduce frequency and strengthen anal sphincter (124). Perianal burning is usually triggered by known foods, such as spices and citrus fruits; such known triggers can be restricted or avoided. There is no specific treatment for burning, but barrier ointment can provide symptomatic relief.

Dyssynergic defecation (DD) or non-relaxing pelvic floor dysfunction is an underdiagnosed pouch disorder (15, 17). It is defined as “the paradoxical contraction and/or impaired relaxation of pelvic floor and anal muscles during defecation” (126). DD can coexist with mechanical and inflammatory pouch disorders. Therefore, it is not unreasonable to assess all IPAA patients presenting with dyschezia for DD, even if initial workup reveals a structural or inflammatory cause (17). When coexisting with inflammatory or mechanical pouch complications, DD can be divided into primary and secondary DD. When DD is the initial trigger leading to fecal stasis and potentially long-standing inflammation as in chronic pouchitis, DD is considered primary. Here, biofeedback therapy targeting DD can improve symptoms, anopouch manometric values, and inflammation. When DD is secondary to chronic pouchitis or pouch outlet stricture or prolapse, it is classified as secondary DD. Here, treating the inflammation or the mechanical disorder can improve symptoms, anopouch manometric values, and inflammation.

There is, at present, no standard criteria for the diagnosis of non-relaxing pelvic floor dysfunction in IPAA patients. Although not validated for IPAA, the same tests used for the diagnosis of DD in patients with an intact colon have been used in IPAA patients using the same normal reference ranges based on healthy controls. These tests include anorectal manometry (ARM) or anopouch manometry, the balloon expulsion test, and barium or magnetic resonance defecography. Abnormal ARM, defined as paradoxical contractions, and failed balloon expulsion were found in one study in 50–60% of patients with functional pouch disorders presenting with dyschezia (15). In another study, a positive balloon expulsion test, defined as >200 g of weight added in the left lateral position or >60 s before balloon expulsion in the seated position, was found in 78% of patients. In contrast, positive ARM, defined as a total of two abnormal ARM values of elevated mean resting anal pressure, reduced pouch–anal gradient, reduced rectal (pouch) pressure, anal relaxation <20%, or an elevated residual anal pressure, was present in only 21% of those with DD. Barium or magnetic resonance defecography can be a useful additional test when balloon expulsion test and ARM are inconclusive, with the added benefit of ruling out pouch outlet obstruction. Finally, since DD that coexists with inflammatory or mechanical pouch complications can be primary or secondary and since there is no simple way of differentiating between the two, assessing response to a trial of biofeedback therapy has been proposed as a non-invasive means of distinguishing the two (19). Primary DD would show manometric and symptomatic response to biofeedback (17). Conversely, those with secondary DD would show symptomatic and manometric response to treating the inflammation with a course of antibiotics or anti-inflammatory or treating the mechanical complication such as stricture (19).

## Pouch Dysplasia and Cancer

### Incidence

The exact incidence of pouch dysplasia and pouch cancer is not clear. In the two largest cohort studies, at 20 years, the incidence of pouch dysplasia was 2.2% in the Cleveland Clinic cohort and 6.9% in the Dutch cohort (127, 128). There are even fewer publications on pouch cancer. The cohort study from The Cleveland Clinic reported a cumulative incidence of cancer of 4.2% at 20 years (127), whereas the Dutch cohort reported a cumulative incidence of 3.2% at 20 years (128). The primary site of dysplasia and cancer is the ATZ or cuff (129).

### Risk Factors for Dysplasia

The single most important risk factor for pouch dysplasia and cancer is colitis-associated neoplasia before colectomy. In The Cleveland Clinic cohort, colitis-associated neoplasia was associated with pouch dysplasia and pouch cancer with hazard ratios of 3.62 (95% CI, 1.59–8.23) and 13.43 (95% CI, 3.96–45.54), respectively. In the Dutch study cohort, colitis-associated neoplasia was similarly associated with dysplasia and cancer of the pouch with hazard ratios of 3.76 (95% CI, 1.39–10.19) and 24.69 (95% CI, 9.61–63.42), respectively (127, 128).

Other risk factors have included concurrent PSC, chronic inflammation of the cuff or the pouch, and mucosal villous atrophy (129). Interestingly, in the Cleveland Clinic cohort, PSC was not shown to be a risk factor, but this might have been due to type II error (127).

### Diagnosis

Pouchoscopy with biopsy is the test of choice for pouch neoplasia surveillance. Neoplastic lesions may appear as depressed, slightly raised lesions or, if advanced, appear mass-like. However, they can also be flat and invisible on endoscopy. In a retrospective study of 11 patients with pouch cancer, 3 (27.3%) had no endoscopically visible lesions at the time of cancer diagnosis (129). The use of narrow band imaging or conventional chromoendoscopy for early detection of pouch neoplasia has not been studied, although their utility in improving polyp detection and colitis-associated neoplasia suggests a potential benefit in at risk patients. Lesions, however, may be endoscopically visible. Until more data are published, we recommend taking at least four or quadrant biopsies from the cuff even if it is normally appearing on white light and chromoendoscopy. As with colitis-associated dysplasia, specimens are best reviewed by an expert gastrointestinal pathologist and any dysplasia confirmed by a second expert pathologist.

### Surveillance

There are no unifying consensus recommendations for pouch neoplasia surveillance. Pouch cancer carries a high mortality (123). Pouchoscopy surveillance can diagnose dysplasia allowing early intervention. Since pouch dysplasia and cancer incidence is low and pouchoscopy and biopsy is somewhat, we support a risk-stratified approach into high, medium, and low risk (129).

#### High Risk

Includes patients with previous colitis-associated neoplasia before or at colectomy, history of indefinite dysplasia of pouch or focal low-grade dysplasia of the pouch. Pouchoscopy is recommended every year.

#### Intermediate Risk

Includes patients with chronic pouchitis, cuffitis, severe mucosal atrophy, previous biopsies showing hyperplastic or serrated changes in the cuff or pouch, concurrent PSC, and family history of colorectal cancer. Pouchoscopy is recommended every 1–2 years.

#### Low Risk

None of the above. Can undergo pouchoscopy every 3 years commencing 10 years after IPAA surgery.

### Treatment

The management of pouch adenocarcinoma is surgical and includes abdominoperineal resection with permanent ileostomy. The need for neo or adjuvant chemotherapy remains unclear due to the rarity of the disease. Because pouch high-grade dysplasia is considered a marker for concurrent or subsequent pouch carcinoma, once confirmed, the recommended treatment is pouch excision (130). Endoscopically resectable pouch low-grade dysplasia should be performed by an experienced endoscopist and followed up closely. If endoscopically invisible or unresectable, pouch low-grade dysplasia should be treated with pouch excision.

## Summary

Quality of life after IPAA surgery is generally good. However, patients can be troubled by pouch-related symptoms and pouch disorders that can be inflammatory, mechanical/surgical, and functional. Maintaining a healthy pouch includes optimizing pouch function, providing advice on a healthy diet and lifestyle, screening for and addressing metabolic complications of IPAA, pouch surveillance, and risk stratification for risk of pouchitis and pouch failure. Patients harboring one or more risk factors for pouchitis can be offered primary prophylaxis. Pouchitis is the most common inflammatory disorder. Primary pouchitis is best classified according to antibiotic response into antibiotic responsive, antibiotic dependent, and antibiotic refractory. This is a spectrum of the same disease. It is predominately microbially mediated early on in acute antibiotic-responsive pouchitis and ends up becoming predominately immune mediated in CARP. Secondary prophylaxis is recommended for recurrent antibiotic-responsive and for antibiotic-dependent pouchitis. Probiotics are first-line secondary as prophylactic agents, followed by the antibiotic rifaximin and then bismuth. Prebiotics such as fibers are best combined with any of the above and delivered in the form of a healthy diet that can be individualized based on patients' tolerance of fermentable fibers. Secondary causes of antibiotic-refractory pouchitis should be ruled out before a diagnosis of CARP is made. Ischemic pouchitis is one of the most common causes. Infections such as CMV and *C. difficile* are associated with fever and night sweats. Other secondary causes include celiac disease, NSAID, and CD of the pouch. Crohn's disease of the pouch can be inflammatory, fibrostenosing, and fistulizing. CARP is best classified as PSC associated, IgG4 associated, and autoimmune. The former two are often associated with PI. PSC-associated CARP and PI can be treated with budesonide or oral vancomycin. Early recognition of IgG4-associated pouchitis minimizes antibiotic use. Budesonide seems to improve inflammation and should be used as first line. Step-up therapy includes immunosuppressive and biologics including anti-TNFs, vedolizumab, and ustekinumab. Autoimmune CARP can be managed in a manner similar to UC. First line includes topical and oral 5ASAs, followed by oral or topical budesonide. There are limited data on the efficacy of immunosuppressives. The current place of immunosuppressives in the treatment algorithm depends on availability and early access to biological agents. Vedolizumab and ustekinumab are the preferred first- and second-line biologics for autoimmune CARP owing to their efficacy, better side effect profile, and low immunogenicity, and need for concomitant immunomodulatory therapy. Anti-TNF should be reserved for autoimmune CARP failing the above and for CD of the pouch. There are no guidelines for the surveillance of pouches for dysplasia. Incidence varies based on a patient's risk. Pouch cancer carries a high mortality. Pouchoscopy surveillance can diagnose dysplasia allowing early intervention. Since incidence is low, however, a risk-stratified approach is recommended.

## Author Contributions

All authors listed have made a substantial, direct and intellectual contribution to the work, and approved it for publication.

### Conflict of Interest

The authors declare that the research was conducted in the absence of any commercial or financial relationships that could be construed as a potential conflict of interest.
